# On the convergence and accuracy of the cardiovascular intrinsic frequency method

**DOI:** 10.1098/rsos.150475

**Published:** 2015-12-16

**Authors:** Peyman Tavallali, Thomas Y. Hou, Derek G. Rinderknecht, Niema M. Pahlevan

**Affiliations:** 1 Aerospace, Division of Engineering and Applied Sciences, California Institute of Technology, 1200 East California Boulevard, MC 205-45, Pasadena, CA 91125, USA; 2 Applied and Computational Mathematics, Division of Engineering and Applied Sciences, California Institute of Technology, 1200 East California Boulevard, MC 9-94, Pasadena, CA 91125, USA; 3 Medical Engineering, Division of Engineering and Applied Sciences, California Institute of Technology, 1200 East California Boulevard, MC 301-46, Pasadena, CA 91125, USA

**Keywords:** intrinsic frequency, instantaneous frequency, pulse wave analysis, cardiovascular disease, ventricular/arterial coupling

## Abstract

In this paper, we analyse the convergence, accuracy and stability of the intrinsic frequency (IF) method. The IF method is a descendant of the sparse time frequency representation methods. These methods are designed for analysing nonlinear and non-stationary signals. Specifically, the IF method is created to address the cardiovascular system that by nature is a nonlinear and non-stationary dynamical system. The IF method is capable of handling specific nonlinear and non-stationary signals with less mathematical regularity. In previous works, we showed the clinical importance of the IF method. There, we showed that the IF method can be used to evaluate cardiovascular performance. In this article, we will present further details of the mathematical background of the IF method by discussing the convergence and the accuracy of the method with and without noise. It will be shown that the waveform fit extracted from the signal is accurate even in the presence of noise.

## 1. Introduction

### 1.1 An introduction to the origins of the intrinsic frequency method

Accessing and using the information hidden in signals requires methods for processing and analysing them. Such methods must be able to denoise and analyse the signal properly in order to process the data. Mathematically, the easiest way to construct a signal processing method is to project the recorded signal on a predetermined algebraic basis or dictionary. A classical method of doing this task is the Fourier transform (FT) method and a more recent one is the wavelet transform (WT) method [1,2]. The FT method is based on the strong assumption of periodicity and lacks the time–frequency localization. On the other hand, the WT method was proposed as a method that incorporates a time–frequency analysis of the signal by constructing a large dictionary of some orthonormal functions.

The FT and WT methods share one common property: decomposition is performed on a predefined basis, which is troublesome if the signal is not stationary. Recently, Huang *et al.* proposed empirical mode decomposition (EMD), a new method of adaptive signal processing [3–5] in which the basis of the projection is adaptive. EMD, which uses multiscale data-driven decompositions called intrinsic mode functions (IMFs), is a step forward in data analysis. It has eliminated most of the issues present in the FT and WT methods [3–5].

In particular, EMD can produce a faithful extraction even if the signal is not periodic, and makes a sparser time–frequency analysis of the data. Projection into a basis is not the ultimate goal in many recently developed signal processing methods. Therefore, researchers have tried to use projections that are as sparse as possible. In other words, it is important to have a representation of the signal in a basis by keeping only a few coefficients containing the pertinent information. In fact, in these methods, one should project the observed signal on a large overdetermined basis (dictionary [6–8]).

Because the IMFs are extracted adaptively from the data in EMD, the final decomposition is in general sparser than FT or WT methods. If the data have a certain frequency scale-separation property, then the extracted IMFs convey certain physical properties of the signal. Unfortunately, the empirical nature of the EMD makes it hard to analyse the results rigorously [9–11]. In order to eliminate this problem, Hou and Shi have proposed a rigorous mathematical system as a counterpart of the EMD method [9–11]. This method is called the sparse time–frequency representation (STFR) method.

All STFR methods are based on the assumption that a relatively large subclass of the oscillatory signals are signals of the form

1.1 x(t)=a(t)cos⁡θ(t),

with only one extrema between the zeros of the signal, in which the envelope is strictly positive, *a*(*t*)>0, and the phase function *θ*(*t*) is a one-to-one, strictly increasing map between the time coordinate, *t*, and the phase coordinate, *θ*. The time derivative of this phase function is called the *instantaneous frequency*. Physically speaking, *θ*(*t*) carries information about the rate of the change of the signal in time. With some abuse of notation, we can say that the STFR methods deal with signals that have both amplitude modulation and frequency modulation. The type of signal in equation (1.1) is called an IMF in STFR terminology. A finite linear combination of a collection of the IMFs is called an intrinsic signal. The goal of the STFR method is to decompose a signal into the sparsest set of IMFs.

A number of methods can extract each IMF from a combination of many IMFs, with different levels of accuracy. Methods that perform such extraction well include, but are not limited to, EMD [4], ensemble EMD [12], optimization-based EMD [13], wavelet [2], STFR [9–11], and synchrosqueezed wavelet transforms [14]. However, when it comes to signals with strong frequency modulation, these methods have difficulties extracting a unique IMF specifically when the data are polluted with noise [9–11,15,16]. Among these methods, the STFR method provides a better physical and mathematical understanding [15].

The intrinsic frequency (IF) method is, in fact, a modified version of the STFR methods [17] that is specialized to analyse certain physiological signals; in this paper, the cardiovascular pulse pressure.

### 1.2 Intrinsic frequency and its clinical importance

When examining the cardiovascular system and the pressure waveforms output by the heart during the cardiac cycle, we see a characteristic signal which is generated by the contraction of the left ventricle, the closure of the aortic valve (dicrotic notch), and the dynamics of the aorta and its branches. In this regard, the phase of the cardiac cycle during left ventricular contraction prior to the dicrotic notch is referred to as systole and the remainder as diastole. By applying the periodic STFR method to the arterial pressure waveforms, we observed that the instantaneous frequency changes its range of oscillation before and after the closure of heart aortic valve (i.e. dicrotic notch); see fig. 6 in [17]. This behaviour was consistently observed across a number of different cardiovascular conditions such as changes in heart rate and aortic rigidity [17,18]. Based on this observation, we proposed a modified version of the STFR to find the dominant frequencies during each respective phase of the cardiac cycle, systole and diastole. We have called this modified version the IF method [17,18].

The IF methodology encompasses the non-stationary dynamics of the cardiovascular system that has been ignored in Windkessel models [19]. Westerhof *et al.* mentioned that ‘Windkessel model is a lumped model of the arterial system or part thereof. Wave transmission and wave travel cannot be studied. Blood flow distribution and changes in the distribution cannot be represented. Effects of local vascular changes, e.g., change in aortic compliance while other vessels are not affected, cannot be studied’ [19]. Therefore, the Windkessel models are extremely limited by their own nature. The IF method, on the other hand, does not make any simplifications or assumptions about the underlying cardiovascular system [17,18]. In this sense, the IF approach and Windkessel approach are fundamentally different. The IF method is constructed based on a more physical model encompassing wave dynamics^1^ and all other dynamical aspects of the cardiovascular system [17,18].

The IF method assumes that because of the dynamics of the heart and aorta, there are two constant dominant frequencies before and after the dicrotic notch. These dominant frequencies have been called IFs. The clinical relevance of the IF method for evaluating cardiovascular performance and detecting cardiovascular disease has been established [17]; e.g. in this paper, we have explicitly shown how *ω*_2_ (see §2) decreases with age (decreasing compliance). Furthermore, the IF method is capable of approximating the left ventricular ejection fraction via non-invasive measurements [26].

Ignoring the effect of Mayer waves, we can assume that the pressure waveform at the entrance of aorta is almost periodic. Using STFR terminology here, we are trying to extract a single IMF from the pressure wave signal. We have shown that this IMF conveys the dynamic characteristics of the heart–aorta system [17,18]. However, the IMF of the pressure wave has a sharp edge at the location of the dicrotic notch (a sudden drop in pressure that occurs at the instant of aortic valve closure). Hence, the definition of an IMF is slightly abused; see [9–11] for a rigorous mathematical definition of an IMF. However, we still call it an IMF. Attempting to extract this IMF using EMD or STFR may fail in some specific cases or produce a blurred extraction, primarily because the change from one frequency regime before the dicrotic notch into another after the closure of the heart valve is accompanied by an abrupt change in frequency of the whole cardiovascular system or by a discontinuity in the first time derivative of the pressure waveform at the dicrotic notch. In the best case, using STFR methods would just capture a vague picture of the instantaneous frequency response of the system, which is good solely for qualitative interpretation and an initial guess for the possible IFs. As a result, we use a modified version of the STFR method that has less mathematical regularity and focuses on the IF rather than on the instantaneous frequency.

This paper illustrates the algorithmic and mathematical properties of the IF algorithm and serves as an extension of previously published work which to date has been presented in a purely physiological context [17,18]. Herein, we demonstrate the convergence of our method with and without noise. Using examples, we express the accuracy of this method both numerically and analytically.

## 2. Intrinsic frequency formulation

It is assumed that before and after the dicrotic notch, we have the following simple waveforms for the general IMF of the aortic pressure wave at time *t*:

2.1 $$s_{i}=a_{i}\cos\omega_{i}t+b_{i}\sin\omega_{i}t+\bar{p},\;i=1,2.$$

This assumption has shown its credibility as an index to characterize the heart and cardiovascular diseases [17,18]. In this formula, *i*=1 corresponds to the behaviour of the IMF before the valve closure, and *i*=2 to the behaviour of the IMF after that. Here, *a*_*i*_,*b*_*i*_ are constants and correspond to the envelopes of the IMF. The constants *ω*_1_,*ω*_2_ correspond to the IFs of the IMF. $\bar{p}$ is the mean pressure during the heart beat period. As the IMF is composed of two different sinusoids, continuous at the dicrotic notch, we can write (2.1) in a more compact form. Take [0,*T*] to be the whole period of the pressure wave and *T*_0_ as the time of the dicrotic notch: 0<*T*_0_<*T*. Also, define the *indicator function* as

$${\text{1}}_{\left[x,y\right)}(t)=\left\{ \begin{array}{ll} 1, & x\leq t<y,\\ 0, & \mathrm{else}. \end{array}\right.$$

Now, the main IMF of the pressure waveform can be expressed as

2.2 $$\begin{array}{rl} S(a_{i},b_{i},\bar{p},\omega_{i};t) & =(a_{1}\cos\omega_{1}t+b_{1}\sin\omega_{1}t+\bar{p}){\text{1}}_{\left[0,T_{0}\right)}(t)\\ & \;+(a_{2}\cos\omega_{2}t+b_{2}\sin\omega_{2}t+\bar{p}){\text{1}}_{\left[T_{0},T\right)}(t). \end{array}$$

If 0≤*t*<*T*_0_, then one would get the part of the IMF corresponding to the action of heart and aorta before the valve closure, i.e. $s_{1}=a_{1}\cos\omega_{1}t+b_{1}\sin\omega_{1}t+\bar{p}$. On the other hand, if *T*_0_≤*t*<*T*, then the part of the IMF that reflects the behaviour of the aorta after the valve closure is depicted by $s_{2}=a_{2}\cos\omega_{2}t+b_{2}\sin\omega_{2}t+\bar{p}$. In general, we are interested in extracting the IMF (2.2) and the corresponding IFs *ω*_1_, *ω*_2_.

At this stage, the goal is to extract the IMF carrying most of the energy and consequently the IFs, *ω*_1_,*ω*_2_, from the observed aortic pressure waveform *f*(*t*). Taking *t* as a continuous variable, one can use least-squares minimization to find the unknowns:

2.3 $$\begin{array}{rl} \underset{a_{i},b_{i},\omega_{i},\bar{p}}{\mathrm{minimize}}\; & ∥f(t)-S(a_{i},b_{i},\bar{p},\omega_{i};t){∥}_{2}^{2}\\ \text{subject to}\; & a_{1}\cos\omega_{1}T_{0}+b_{1}\sin\omega_{1}T_{0}=a_{2}\cos\omega_{2}T_{0}+b_{2}\sin\omega_{2}T_{0},\\ \; & a_{1}=a_{2}\cos\omega_{2}T+b_{2}\sin\omega_{2}T. \end{array}$$

In this optimization problem, $∥g(t){∥}_{2}^{2}=\int_{0}^{T}|g(t){|}^{2}\;dt$. The first linear condition in this optimization enforces the continuity of the extracted IMF at the dicrotic notch. The second one imposes the periodicity. In practice, as the data are sampled on discrete temporal points, one must solve the discrete version of (2.4). Assume that the data are sampled on time instances 0=*t*_1_<*t*_2_<⋯<*t*_*n*_=*T*, then one can convert problem (2.4) into a discrete least-squares of the form

2.4 $$\begin{array}{rl} \underset{a_{i},b_{i},\omega_{i},\bar{p}}{\text{minimize}}\; & \sum_{j=1}^{n}{\left(f(t_{j})-S(a_{i},b_{i},\bar{p},\omega_{i};t_{j})\right)}^{2}\\ \text{subject to}\; & a_{1}\cos\omega_{1}T_{0}+b_{1}\sin\omega_{1}T_{0}=a_{2}\cos\omega_{2}T_{0}+b_{2}\sin\omega_{2}T_{0},\\ & a_{1}=a_{2}\cos\omega_{2}T+b_{2}\sin\omega_{2}T. \end{array}$$

Note that problem (2.5) is not convex. Therefore, we used a brute-force algorithm to solve it.

## 3. Algorithm

In algorithm 1, we break down the problem into a convex part and a global domain search [27]. The domain search part is the brute-force part of the algorithm. For this algorithm, the frequency domain is

3.1 $$D_{\mathrm{fr}}=\left\{\left(\omega_{1},\omega_{2}\right)|0<\omega_{1}\leq C,0<\omega_{2}\leq C\right\},$$

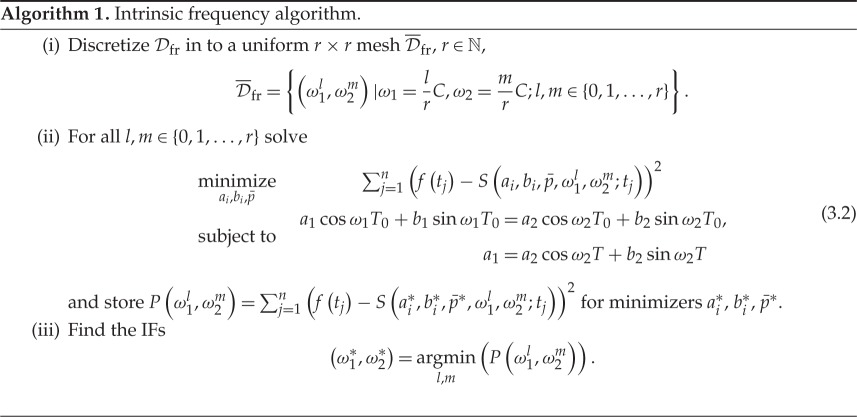


which is characterized by a constant parameter, *C*, that depends on the physics of the problem. The basic idea behind algorithm 1 is to freeze (*ω*_1_,*ω*_2_), solve (2.5), and find the minimum of the function

$$P(\omega_{1},\omega_{2})=\sum_{j=1}^{n}{\left(f(t_{j})-S(a_{i}^{*},b_{i}^{*},{\bar{p}}^{*},\omega_{i};t_{j})\right)}^{2},$$

where $a_{i}^{*}$, $b_{i}^{*}$, ${\bar{p}}^{*}$ are the values upon which (2.5) is minimized for a fixed vector (*ω*_1_,*ω*_2_). We collect all possible values of *P*(*ω*_1_,*ω*_2_) and find the minimum of them among (*ω*_1_,*ω*_2_). The minimizer of *P* over (*ω*_1_,*ω*_2_) would then be the IFs that is the solution of the optimization problem.

The second step of algorithm 1 is just solving a linearly constrained least-squares algorithm on *a*_*i*_,*b*_*i*_ and $\bar{p}$. This brute-force algorithm can also be made parallel computationally, because step (ii) can be solved separately for different (*l*,*m*) pairs.

## 4. Convergence analysis

In this section, we analyse the convergence properties of algorithm 1. In order to discuss the algorithm’s convergence and accuracy, we need the following lemma [28] and theorem.

**Lemma 4.1**

*The minimum of a function can first be found over a few variables and then over the remaining ones*:

$$\underset{x,y}{\inf}\;f(x,y)=\underset{x}{\inf}\left(\underset{y}{\inf\;f(x,y)}\right).$$

Lemma 4.1 allows us to first find the minimization (2.5) on $a_{i},b_{i},\bar{p}$ and then on *ω*_1_,*ω*_2_. Further, we need to make sure that the second step of the algorithm has a unique minimizer, which can be provided by the following theorem [29].

**Theorem 4.2**

*If* $A\in R^{m\times n},$ $C\in R^{p\times n},$ $x\in R^{n},$ $b\in R^{m},$ $d\in R^{p}$, *where p*≤*n, n*≤*m*+*p and* $\left[\begin{array}{c} A\\ C \end{array}\right]$ *is of full column rank, then the optimization problem
*

$$\begin{array}{rl} \underset{x}{\mathrm{minimize}}\; & ∥Ax-b{∥}_{2}^{2}\\ \mathrm{subject to}\; & Cx=d \end{array}$$

*has a unique solution.*

As the algorithm freezes the frequency parameters (*ω*_1_,*ω*_2_) and then solves a least-squares problem on other variables, we can form a notation similar to that used in theorem 4.2. Take the matrix *A* to be

$$A=\left(\begin{array}{ccccc} \cos\omega_{1}t_{1} & \sin\omega_{1}t_{1} & 0 & 0 & 1\\ \cos\omega_{1}t_{2} & \sin\omega_{1}t_{2} & 0 & 0 & 1\\ \vdots & \vdots & \vdots & \vdots & \vdots \\ \cos\omega_{1}t_{n_{0}} & \sin\omega_{1}t_{n_{0}} & 0 & 0 & 1\\ 0 & 0 & \cos\omega_{2}t_{n_{0}+1} & \sin\omega_{2}t_{n_{0}+1} & 1\\ \vdots & \vdots & \vdots & \vdots & \vdots \\ 0 & 0 & \cos\omega_{2}t_{n} & \sin\omega_{2}t_{n} & 1 \end{array}\right),$$

and the matrix *C* and vector *x* to be

$$C=\left(\begin{array}{ccccc} \cos\omega_{1}t_{n_{0}} & \sin\omega_{1}t_{n_{0}} & -\cos\omega_{2}t_{n_{0}} & -\sin\omega_{2}t_{n_{0}} & 0\\ 1 & 0 & -\cos\omega_{2}t_{n} & -\sin\omega_{2}t_{n} & 0 \end{array}\right),\;x=\left(\begin{array}{c} a_{1}\\ b_{1}\\ a_{2}\\ b_{2}\\ \bar{p} \end{array}\right).$$

Sample points *t*_1_,…,*t*_*n*_0__ correspond to the trend before the dicrotic notch and *t*_*n*_0_+1_,…,*t*_*n*_ to the points after it. The vector *b* would be the observed sampled signal, {*b*}_*j*_=*f*(*t*_*j*_), *j*=1,2,…,*n*. Finally, *d*=**0** is enforcing the periodicity and continuity of the waveform when imposed on the right-hand side of *Cx*=*d*. These matrices and vectors satisfy the conditions of theorem 4.2. Hence, the second step of the algorithm always has a unique solution. This fact, combined with lemma 4.1, guarantees that algorithm 1 always has at least one unique solution for the IF minimization problem (2.5).

### 4.1 Noise-free condition

#### 4.1.1 Existence

Assume there is no noise in the observation and the observed signal is exactly of type (2.2). As a result, the signal can be expressed as $f=\bar{A}\bar{x},\bar{C}\bar{x}=0$ for some $\left({\bar{\omega }}_{1},{\bar{\omega }}_{2}\right)$ and $\bar{x}$. If there is a well resolved ${\bar{D}}_{\mathrm{fr}}$, then for some *l*, *m*, one would obtain $\left(\omega_{1}^{l},\omega_{2}^{m})=({\bar{\omega }}_{1},{\bar{\omega }}_{2}\right)$. At these specific frozen frequencies, the solution of the second step of the algorithm is nothing but $\bar{x}$, based on theorem 4.2. More specifically, the problem that is being solved at this step is

$$\begin{array}{rl} \underset{x}{\text{minimize}}\; & ∥\bar{A}x-\bar{A}\bar{x}{∥}_{2}^{2}\\ \text{subject to}\; & \bar{C}x=0. \end{array}$$

Therefore, considering the definition of *P*(*ω*_1_,*ω*_2_) and lemma 4.1, it is guaranteed that there exists at least one minimizer.

#### 4.1.2 Uniqueness

Furthermore, this minimizer is unique. In fact, if there exists another set of *x* and (*ω*_1_,*ω*_2_) as the solution of problem (2.5), namely ${\bar{x}}^{\prime }$ and $\left({\bar{\omega }}_{1}^{\prime },{\bar{\omega }}_{2}^{\prime }\right)$, then the two trends, $\bar{A}\bar{x}$ and ${\bar{A}}^{\prime }{\bar{x}}^{\prime }$, arising from these parameters must be equal. For a finely sampled observation *f*, equality of these trends implies the equality of the parameters ${\bar{x}}^{\prime }=\bar{x}$, $\left({\bar{\omega }}_{1}^{\prime },{\bar{\omega }}_{2}^{\prime })=({\bar{\omega }}_{1},{\bar{\omega }}_{2}\right)$. In short, we can state the following theorem.

**Theorem 4.3**

*In the absence of noise, if the observed signal is of the form (*2.2*), for a well resolved* ${\bar{D}}_{\mathrm{fr}},$ *algorithm 1 finds the unique minimizer of
*

$$\begin{array}{rl} \underset{a_{i},b_{i},\omega_{i},\bar{p}}{\text{minimize}}\; & \sum_{j=1}^{n}{\left(f(t_{j})-S(a_{i},b_{i},\bar{p},\omega_{i};t_{j})\right)}^{2}\\ \text{subject to}\; & a_{1}\cos\omega_{1}T_{0}+b_{1}\sin\omega_{1}T_{0}=a_{2}\cos\omega_{2}T_{0}+b_{2}\sin\omega_{2}T_{0},\\ \; & a_{1}=a_{2}\cos\omega_{2}T+b_{2}\sin\omega_{2}T. \end{array}$$

### 4.2 Noisy measurements

Assume here that the IMF (2.2) is polluted with noise that is independent of the IMF itself. In other words, taking the noise to be *ε*, $f=\bar{A}\bar{x}+\varepsilon ,\bar{C}\bar{x}=0$. Here, the algorithm will not extract the exact values of $\left({\bar{\omega }}_{1},{\bar{\omega }}_{2}\right)$ and $\bar{x}$, but it is possible to find an error bound on the distance between the extracted and the true IMF. If x* and $\left(\omega_{1}^{*},\omega_{2}^{*}\right)$ are the extracted values by the algorithm, then one can write

$$∥A^{*}x^{*}-\bar{A}\bar{x}-\varepsilon {∥}_{2}\leq ∥Ax-\bar{A}\bar{x}-\varepsilon {∥}_{2}\leq ∥\varepsilon {∥}_{2}$$

The first inequality comes from the fact that any set of *x* and (*ω*_1_,*ω*_2_), where *Cx*=0, is a feasible point; consequently, the second inequality follows if *x* and (*ω*_1_,*ω*_2_) are assigned the values of $\bar{x}$ and $\left({\bar{\omega }}_{1},{\bar{\omega }}_{2}\right)$. Now, using the triangle inequality one can show

$$∥A^{*}x^{*}-\bar{A}\bar{x}{∥}_{2}\leq ∥A^{*}x^{*}-\bar{A}\bar{x}-\varepsilon {∥}_{2}+∥\varepsilon {∥}_{2}\leq 2∥\varepsilon {∥}_{2}.$$

Using this, the following theorem can be proposed.

**Theorem 4.4**

*In the presence of a trend-independent noise in (*2.2*), for a well resolved* ${\bar{D}}_{\mathrm{fr}},$ *algorithm 1 finds the minimizer of (*2.5*) with an error having at most the same order as the noise.*

Remember that in this theorem, a well-resolved ${\bar{D}}_{\mathrm{fr}}$ is a discretized domain in which the distance between two adjacent mesh points is sufficiently small. In this theorem, it means that it is a continuum, and in practice, we have found that the maximum distance of 0.001 between the mesh points can construct a well-resolved ${\bar{D}}_{\mathrm{fr}}$.

How much the solution of the noisy problem differs from the real solution depends on the noise level. If the noise level ∥*ε*∥ is sufficiently small, then the distance between x*, $\left(\omega_{1}^{*},\omega_{2}^{*}\right)$ and $\bar{x}$, $\left({\bar{\omega }}_{1},{\bar{\omega }}_{2}\right)$ is also of *O*(∥*ε*∥); see [30]. In practice, because the 2-norm of the trend is large compared with the noise level, the relative error in finding the trend is extremely low. In mathematical terms, we have

$$\frac{∥A^{*}x^{*}-\bar{A}\bar{x}{∥}_{2}}{∥\bar{A}\bar{x}{∥}_{2}}\leq 2\frac{∥\varepsilon {∥}_{2}}{∥\bar{A}\bar{x}{∥}_{2}}.$$

So, in general, algorithm 1 enables us to extract the IFs with a bounded error, even in the presence of noise perturbation. In real data, where a lot of reflected waves are superposed with the heart–aorta wave system, the signal is, in general, a combination of multiple IMFs. Usually, these waves have higher frequencies compared with the main IMF. This point will be made clearer in the next subsection.

### 4.3 Higher-order intrinsic mode functions

For the sake of simplicity, we assume that the added IMFs are of high frequency and that time is a continuous variable and the signal is not sampled on discrete points (it is a continuous variable). Take the recorded signal to be

4.1 $$g(t)=\bar{S}(t)+D_{M}(t),$$

where $\bar{S}(t)$ is the IMF of form (2.2), and *D*_*M*_(*t*) is a combination of IMFs with higher frequencies compared with $\bar{S}(t)$. Without loss of generality, take *D*_*M*_(*t*) to have a Fourier series of the form

4.2 $$D_{M}(t)=\sum_{n>M}\left(A_{n}\cos\frac{2\pi nt}{T}+B_{n}\sin\frac{2\pi nt}{T}\right).$$

Implicitly, we have assumed that the added IMFs are of high-frequency nature. Having this terminology in mind, we can state the following theorem.

**Theorem 4.5**

The optimum curve S*(t), which is the solution of the minimization problem

4.3 minimizeS(t)∥S(t)−S¯(t)−DM(t)∥L22subject toS(t) is continuous at T0,S(t) is periodic,

*satisfies
*

4.4 $$∥S^{*}(t)-\bar{S}(t){∥}_{L^{2}}\leq \frac{8∥D_{M}^{\left(m\right)}(t){∥}_{L^{1}}}{(m-1)\sqrt{T}{\left(M+1\right)}^{m-1}},$$

*provided that D*_*M*_(*t*)∈*C*^*m*^, *and m*≥2.^2^

**Proof.**

As $\bar{S}(t)$ is a feasible point, the minimizer S*(*t*) of (4.3) must satisfy

4.5 $$∥S^{*}(t)-\bar{S}(t)-D_{M}(t){∥}_{L^{2}}^{2}\leq ∥\bar{S}(t)-\bar{S}(t)-D_{M}(t){∥}_{L^{2}}^{2}=∥D_{M}(t){∥}_{L^{2}}^{2}.$$

Define the Fourier series of $S^{*}(t)-\bar{S}(t)$ as

$$S^{*}(t)-\bar{S}(t)=\frac{1}{2}\Delta a_{0}+\sum_{n=1}^{\infty }\left(\Delta a_{n}\cos\frac{2\pi nt}{T}+\Delta b_{n}\sin\frac{2\pi nt}{T}\right).$$

Hence, we have

$$\begin{array}{rl} S^{*}(t)-\bar{S}(t)-D_{M}(t) & =\frac{1}{2}\Delta a_{0}+\sum_{n=1}^{M}\left(\Delta a_{n}\cos\frac{2\pi nt}{T}+\Delta b_{n}\sin\frac{2\pi nt}{T}\right)\\ & \;+\sum_{n=M+1}^{\infty }\left((\Delta a_{n}-A_{n})\cos\frac{2\pi nt}{T}+(\Delta b_{n}-B_{n})\sin\frac{2\pi nt}{T}\right). \end{array}$$

Using Parseval’s identity^3^ and (4.5) gives

$$\begin{array}{rl} ∥S^{*}(t)-\bar{S}(t)-D_{M}(t){∥}_{L^{2}}^{2} & =∥S^{*}(t)-\bar{S}(t){∥}_{L^{2}}^{2}+∥D_{M}(t){∥}_{L^{2}}^{2}\\ & \;-2T\sum_{n=M+1}^{\infty }(\Delta a_{n}A_{n}+\Delta b_{n}B_{n})\leq ∥D_{M}(t){∥}_{L^{2}}^{2}. \end{array}$$

Simplifying and using triangle inequality result in

4.6 $$∥S^{*}(t)-\bar{S}(t){∥}_{L^{2}}^{2}\leq 2T\sum_{n=M+1}^{\infty }(|\Delta a_{n}∥A_{n}|+|\Delta b_{n}∥B_{n}|).$$

Because $S^{*}(t)-\bar{S}(t)$ is continuous, $|\Delta a_{n}|,|\Delta b_{n}|\leq ∥S^{*}(t)-\bar{S}(t){∥}_{L^{1}}/T$. Using the Cauchy–Schwartz inequality gives $|\Delta a_{n}|,|\Delta b_{n}|\leq ∥S^{*}(t)-\bar{S}(t){∥}_{L^{2}}/\sqrt{T}$. On the other hand, as *D*_*M*_(*t*)∈*C*^*m*^, then we get $|A_{n}|,|B_{n}|\leq 2(∥D_{M}^{\left(m\right)}(t){∥}_{L^{1}}/Tn^{m})$. Using these estimates and bounding the sum by an integral, (4.6) will result in

$$∥S^{*}(t)-\bar{S}(t){∥}_{L^{2}}\leq \frac{8∥D_{M}^{\left(m\right)}(t){∥}_{L^{1}}}{(m-1)\sqrt{T}{\left(M+1\right)}^{m-1}}.$$

 □

**Remark 4.6**

The bound (4.4) shows that as the minimum frequency *M* of the IMFs increases, then the optimum curve S*(*t*) and the true curve $\bar{S}(t)$ get closer. This bound is in fact a measure of the ‘scale separation condition’ mentioned in [9–11]. In simple words, if the IMFs are orthogonal to the original IMF, $\bar{S}(t)$, then the extracted optimum curve S*(*t*) is almost the true IMF $\bar{S}(t)$. Hence, the frequencies found in S*(*t*) are close to true IF values. Note that, in deriving this bound, we have not used the structure of the main IMF. Therefore, this bound is in general an orthogonality condition. In practice, the bounds are even smaller than (4.4). This means that the algorithm performs better than the error estimate.

## 5. Results

Here, we will show how the results of the proposed algorithm 1 perform using synthetic and clinical examples.^4^

**Example 5.1**

**Figure 1.**
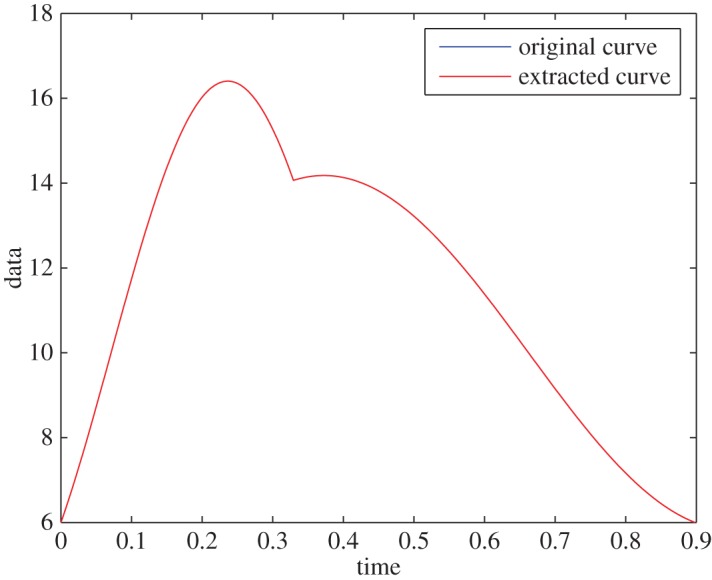
Synthetic data, without noise and with a well-resolved domain.

Assume that the signal has intrinsic frequencies of *ω*_1_= 9.5 rad s^−1^ and *ω*_2_=5.42 rad s^−1^. The envelope of the first part of the trend is taken to be $\sqrt{41}$, and the envelope of the second part is taken in a way that it matches a signal of period 0.898 s and a dicrotic notch at *T*_0_=0.3293 s. The mean value of the signal is $\bar{p}=10$. ${\bar{D}}_{\mathrm{fr}}$ is defined in a way that the resolution of the frequency domain, $\min_{l\neq m}(|\omega_{1}^{l}-\omega_{1}^{m}|,|\omega_{2}^{l}-\omega_{2}^{m}|)$, is 0.08 rad s^−1^. For this well-resolved domain, the extraction is accurate up to machine error precision: the curves are indistinguishable (figure 1). The IFs are found with no error.

**Example 5.2**

**Figure 2.**
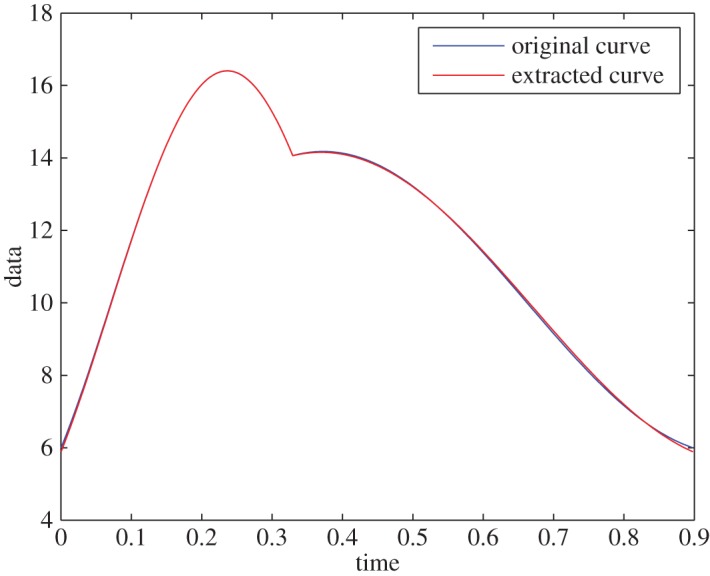
Synthetic data, without noise and without a well-resolved domain.

To test the algorithm for a case in which ${\bar{D}}_{\mathrm{fr}}$ is not well resolved, we use the signal from example 5.1 and define ${\bar{D}}_{\mathrm{fr}}$, so that the resolution of the frequency domain is 0.1 rad s^−1^. This resulted in a faithful extraction of the curve, with less than 0.4% relative error^5^ (figure 2). The IFs are $\omega_{1}^{*}=9.4\;{\mathrm{rad}\;s}^{-1}$ and $\omega_{2}^{*}=5.2$ rad s^−1^, which have less than 5% relative error (less than 0.47 rads^−1^ in absolute measure).

In example 5.3, we will investigate the effect of noise on the extracted IFs. Here, ${\bar{D}}_{\mathrm{fr}}$ is well resolved.

**Example 5.3**

**Figure 3.**
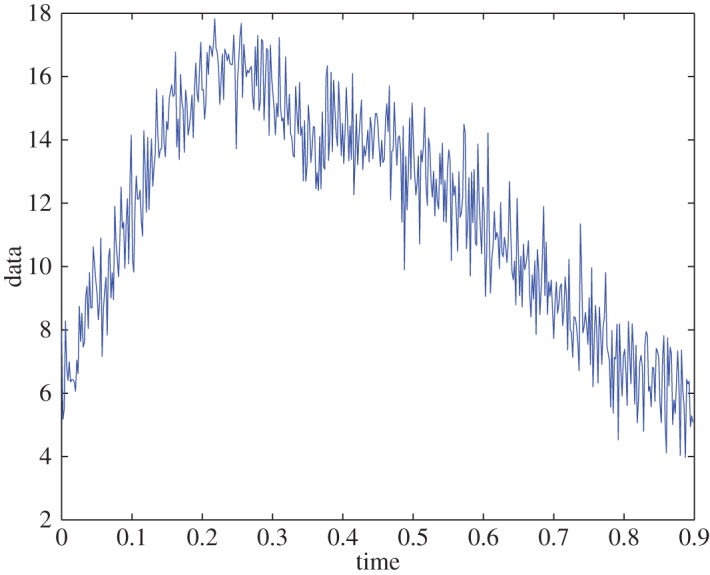
Original noisy data.

**Figure 4.**
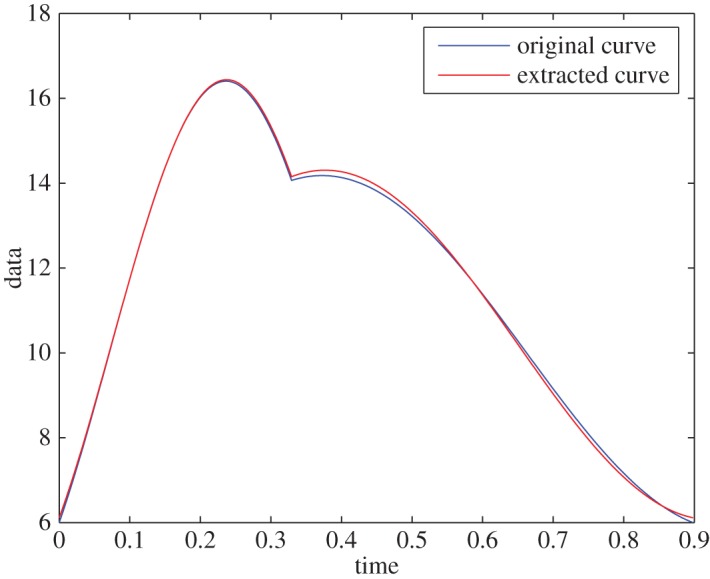
Extracted curve versus the original curve in a noisy environment.

To show the stability of the algorithm and to test the effect of noise on a signal with well-resolved ${\bar{D}}_{\mathrm{fr}}$, we use the same signal and define ${\bar{D}}_{\mathrm{fr}}$, so that the resolution of the frequency domain is 0.027 rad s^−1^. The signal is polluted with normal random Gaussian (mean zero and variance one) noise (figure 3) and the relative error in extraction is less than 0.7% (figure 4). The extracted IFs were $\omega_{1}^{*}=9.61\;\mathrm{rad}\;s^{-1}$ and $\omega_{2}^{*}=5.74\;\mathrm{rad}\;s^{-1}$, which have relative error of less than 6%. This example shows the stability of the algorithm.

**Example 5.4**

**Figure 5.**
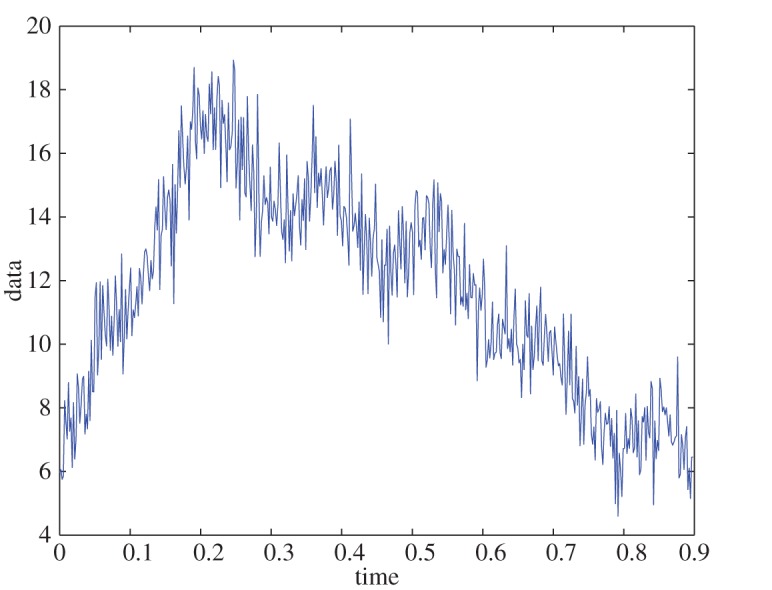
Synthetic trend plus IMF and noise.

**Figure 6.**
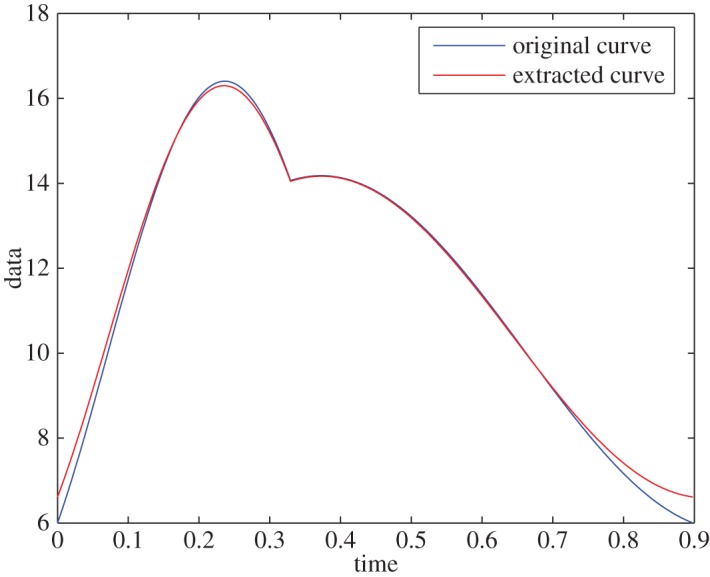
Extracted trend for the IMF and noise case.

(a) To synthetically examine the effect of adding an IMF to the main IMF, a simple sine wave of form sin⁡(20πt/T) is added to the same signal used in previous examples. The extracted IFs are again accurate. In fact, if ${\bar{D}}_{\mathrm{fr}}$ has a resolution of 0.027 rad s^−1^, the error in extracted IFs is zero up to three digits of accuracy. With a ${\bar{D}}_{\mathrm{fr}}$ resolution of 0.25 rad s^−1^, the relative error was at most 8%. These examples show that the algorithm works better than the bound provided by (4.4).
(b) To test whether a noisy observation with an added low frequency IMF would be still a tractable problem for the algorithm, we take the IMF from example 5.1 and add sin⁡(4πt/T)+N(0,1) (figure 5). Here, N(0,1) is the white (Gaussian) noise with mean zero and variance one. For a ${\bar{D}}_{\mathrm{fr}}$ resolution of 0.08 rad s^−1^, the extracted IFs are $\omega_{1}^{*}=9.66\;\mathrm{rad}\;s^{-1}$ and $\omega_{2}^{*}=6.14\;\mathrm{rad}\;s^{-1}$ and the maximum relative error in extracted IFs is less than 14%. If a higher resolution is used, the results are much better. For example, for a resolution of 0.027 rad s^−1^, the extracted IFs are $\omega_{1}^{*}=9.61\;\mathrm{rad}\;s^{-1}$ and $\omega_{2}^{*}=5.79\;\mathrm{rad}\;s^{-1}$, having a maximum relative error of just 7% (figure 6). The curve extraction has a relative error of 2%.

**Example 5.5**

**Figure 7.**
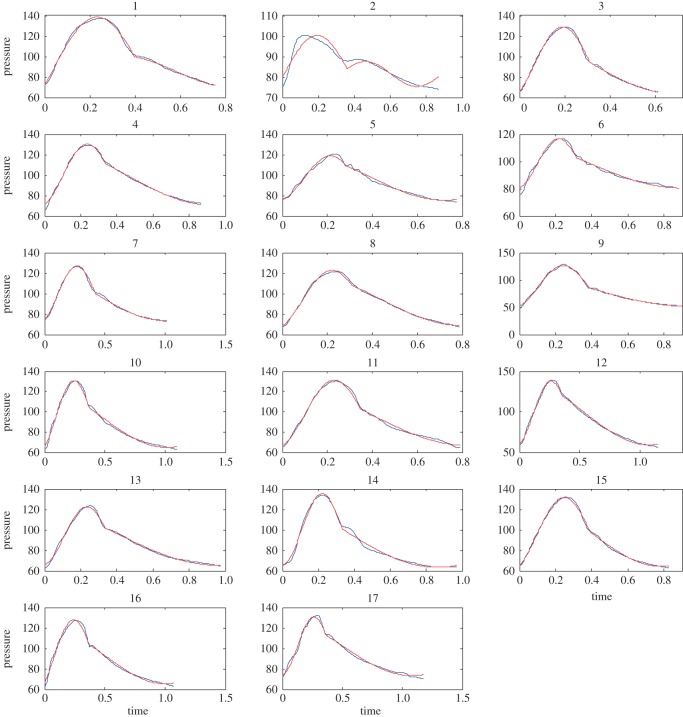
Recorded clinical data. The data were collected from the ascending aorta of subjects using a catheter (blue curves). The data were then analysed using the IF algorithm (red curves).

We have tested the performance of the IF algorithm on clinical data [17,18]. Here, we present the details of some of the cases presented in that work, in figure 7. The data are collected from the ascending aorta of subjects using a catheter. This original recorded signal is shown in blue. The extracted IMFs are represented in red. In all cases, the dicrotic notch was identified based on eye inspection by an expert in the field. One can observe how the IMF is faithfully extracted from the data in most cases. The extraction in case 2, in figure 7, needs more attention: as can be seen from figure 7, if the rising portion in systole is shorter in time than the falling part (effect of the second IMF), the extracted IMF is not as good as other cases. We believe this issue can be addressed by some modifications in the current IF method. We will address these special cases in a future work.

**Example 5.6**

**Figure 8.**
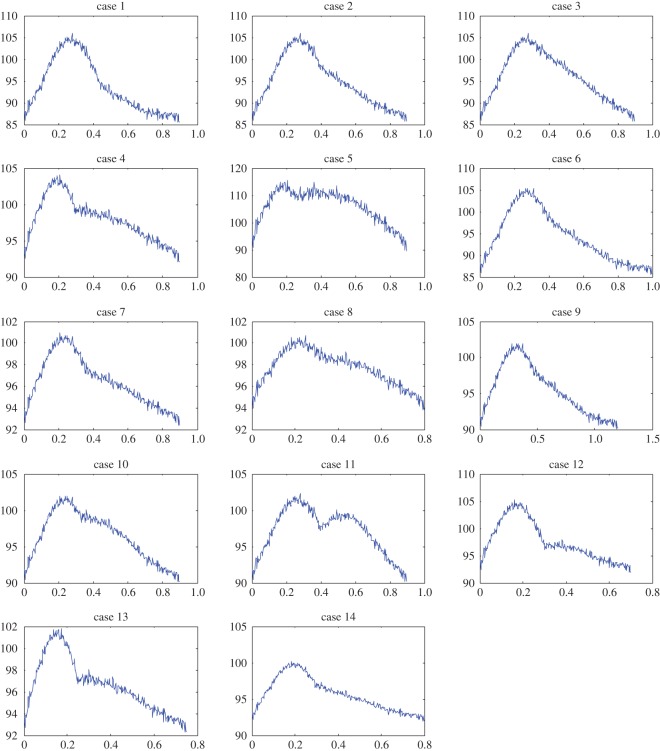
Synthetic data with noise. The data were created using deterministic functions with added noise.

**Figure 9.**
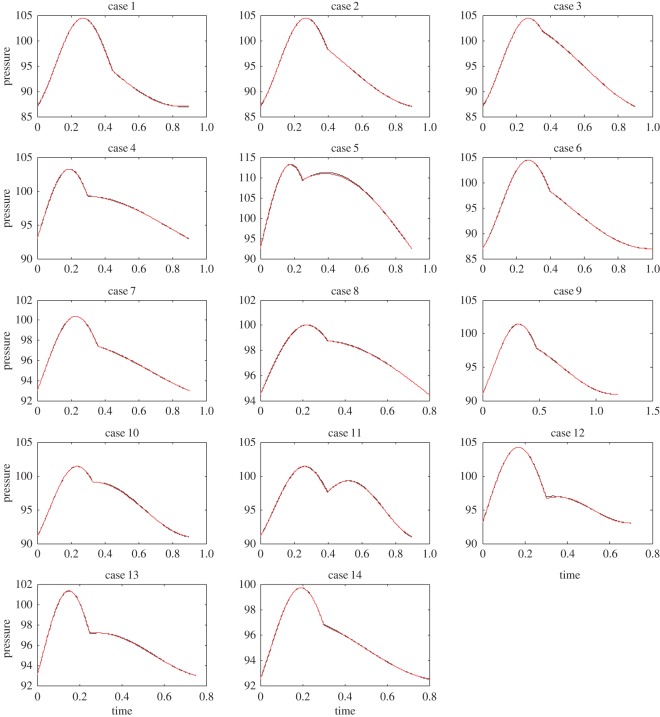
Synthetic data for IMF extraction. The original signal without the noise is shown in black. The extracted IMF using the IF algorithm is shown in red.

In this example, we intend to experimentally observe the sensitivity of the algorithm in the presence of noise, as a preliminary work (figure 8). The data were created using a deterministic form like (2.1). Then, noise is added to the signal based on the norm of the oscillatory part of the signal. From figure 8, one can observe that the data are greatly affected by noise. The dicrotic notches in these cases were predefined. The results of the IF algorithm are shown in figure 9. Figure 9 shows the accuracy of the algorithm in the presence of noise. Table 1 includes the extracted IF values for these cases. A more rigorous statistical analysis, similar to this example, on the sensitivity of our method will be done in our future work.

**Table 1.** The results of the IF algorithm: original frequencies in units of rad s^−1^ are shown as *ω*_1_ and *ω*_2_. The extracted values, $\omega_{1}^{*}$, $\omega_{2}^{*}$, and the absolute errors, $|\omega_{1}-\omega_{1}^{*}|$, $|\omega_{2}-\omega_{2}^{*}|$, are also expressed.

	*ω*_1_	$\omega_{1}^{*}$	$|\omega_{1}-\omega_{1}^{*}|$	*ω*_2_	$\omega_{2}^{*}$	$|\omega_{2}-\omega_{2}^{*}|$
case 1	9.5	9.6105	0.1105	3.42	3.5881	0.1681
case 2	9.5	9.6105	0.1105	3.42	3.4412	0.0212
case 3	9.5	9.4636	0.0364	3.42	3.4412	0.0212
case 4	9.5	8.8761	0.6239	3.42	2.7068	0.7132
case 5	9.5	8.1417	1.3583	3.42	2.8537	0.5663
case 6	9.5	9.4636	0.0364	3.42	3.4412	0.0212
case 7	8.5	8.4354	0.0646	3.42	2.8537	0.5663
case 8	7.5	6.8197	0.6803	3.42	2.8537	0.5663
case 9	7	7.1134	0.1134	3.42	3.2944	0.1256
case 10	9.5	9.4636	0.0364	5.42	5.2039	0.2161
case 11	8.5	8.5823	0.0823	7.42	7.4072	0.0128
case 12	10.5	10.4919	0.0081	8.42	9.4636	1.0436
case 13	12.5	12.5483	0.0483	6	5.7915	0.2085
case 14	11	10.7856	0.2144	4	4.3226	0.3226

## 6. Discussion and conclusion

In this article, we sketched the algorithmic and mathematical properties of the IF algorithm. We have demonstrated the convergence of our method with and without noise. Using examples, we showed the accuracy of this method that is in accord with the mathematical accuracy bounds presented in the paper. The convergence and stability of the algorithm, combined with the physiological intuition of the heart–aorta model [17,18], make it a suitable method for rigorous pulse pressure analysis.

In the clinical data examples, we could clearly show that the IF method can capture the behaviour of the pulse pressure waveform with good accuracy. As these examples show, the characteristics of an aortic waveform (figure 7) can be expressed with only a few parameters $\left(a_{1},a_{2},b_{1},b_{2},\bar{p},\omega_{1},\omega_{2}\right)$. In other words, the IF can work as a dimensionality reduction method to accurately capture the complex physics of such waveforms using a limited set of parameters. Furthermore, the clinical data have confirmed that the IFs, *ω*_1_ and *ω*_2_, are the most physiologically relevant parameters [17,18]. The other five parameters are auxiliary parts of the mathematical construction of the method.

The IF is superior to the FT representation because the IF can localize the frequency behaviour of the waveform. For example, the FT is not capable of recognizing the time instance of the harmonics, whereas the IF does not suffer from this issue. In fact, the IF is constructed from a nonlinear and non-stationary point of view to dynamical systems [17,18]. The FT, by contrast, assumes the system is both linear and stationary in its approach. From a physiological point of view, there are two main non-stationary effects in the heart and vascular system. The first is heart rate variability. The second is the closure of the aortic valve which depends on dynamics of both the heart and vascular system. The localization behaviour of the IF allows us to clearly separate these non-stationary effects. Physiologically speaking, this means the IF can distinguish between waveform abnormalities originating from the heart versus the aortic and arterial system [17,18]. Last but not least, the FT represents the pulse pressure with at least five linear harmonics distributing the energy among them. This distribution of energy loses critical information. On the contrary, the IF represents the pulse pressure with only two nonlinear harmonics (*ω*_1_ and *ω*_2_). Hence, the IF is a simpler, more meaningful concept that is capable of accurately quantifying the complex system dynamics of heart and aorta.

## Footnotes

1 The importance of the wave dynamics has also been addressed in the literature [20–25].

2 This bound can be made sharper if *D*_*M*_(*t*)∈*C*^*m*^ and $D_{M}^{\left(m+1\right)}(t)\in L_{\left(0,T\right)}^{2}$. Here the *p*-norm is defined as $∥\vartheta (t){∥}_{L^{p}}={\left(\int_{0}^{T}|\vartheta (t){|}^{p}\;dt\right)}^{1/p}$ for *p*≥1.

3 In an inner-product vector space with an orthonormal basis *B*, for any vector *x* in this space, we have $∥x{∥}^{2}=\sum_{e\in B}|\langle x,e\rangle {|}^{2}$.

4 The datasets supporting this article have been uploaded as part of the electronic supplementary material [31].

5 Relative error for the signal is defined as $∥S^{*}(t)-\bar{S}(t){∥}_{L^{2}}/∥\bar{S}(t){∥}_{L^{2}}.$

## Data accessibility

Data used in this study can be downloaded from http://dx.doi.org/10.5061/dryad.21q9m.

## References

[1] Blatter C 2002 Wavelets: a primer. New York, NY: Taylor & Francis.

[2] Mallat SG 1999 A wavelet tour of signal processing. San Diego, CA: Academic Press.

[3] Huang NE, Shen SS 2005 Hilbert–Huang transform and its applications, vol. 5 Singapore: World Scientific.

[4] Huang NE, Shen Z, Long SR, Wu MC, Shih HH, Zheng Q, Yen NC, Tung CC, Liu HH 1998 The empirical mode decomposition and the Hilbert spectrum for nonlinear and non-stationary time series analysis. Proc. R. Soc. Lond. A 454, 903–995 (doi:10.1098/rspa.1998.0193)

[5] Huang NE, Wu Z, Long SR, Arnold KC, Chen X, Blank K 2009 On instantaneous frequency. Adv. Adapt. Data Anal. 1, 177–229. (doi:10.1142/S1793536909000096)

[6] Candes EJ, Tao T 2006 Near-optimal signal recovery from random projections: universal encoding strategies? IEEE Trans. Inf. Theory 52, 5406–5425. (doi:10.1109/TIT.2006.885507)

[7] Chen SS, Donoho DL, Saunders MA 1998 Atomic decomposition by basis pursuit. SIAM J. Sci. Comput. 20, 33–61. (doi:10.1137/S1064827596304010)

[8] Mallat SG, Zhang Z 1993 Matching pursuits with time–frequency dictionaries. IEEE Trans. Signal Process. 41, 3397–3415. (doi:10.1109/78.258082)

[9] Hou TY, Shi Z, Tavallali P 2014 Convergence of a data-driven time–frequency analysis method. Appl. Comput. Harmon. Anal. 37, 235–270. (doi:10.1016/j.acha.2013.12.004)

[10] Hou TY, Shi Z 2011 Adaptive data analysis via sparse time–frequency representation. Adv. Adapt. Data Anal. 3, 1–28. (doi:10.1142/S1793536911000647)

[11] Hou TY, Shi Z 2013 Data-driven time–frequency analysis. Appl. Comput. Harmon. Anal. 35, 284–308. (doi:10.1016/j.acha.2012.10.001)

[12] Wu Z, Huang NE 2009 Ensemble empirical mode decomposition: a noise-assisted data analysis method. Adv. Adapt. Data Anal. 1, 1–41. (doi:10.1142/S1793536909000047)

[13] Huang B, Kunoth A 2012 An optimization based empirical mode decomposition scheme. J. Comput. Appl. Math. 240, 174–183. (doi:10.1016/j.cam.2012.07.012)

[14] Daubechies I, Lu J, Wu H 2011 Synchrosqueezed wavelet transforms: an empirical mode decomposition-like tool. Appl. Comput. Harmon. Anal. 30, 243–261. (doi:10.1016/j.acha.2010.08.002)

[15] Hou TY, Shi Z, Tavallali P 2013 Sparse time frequency representations and dynamical systems. (http://arxiv.org/abs/1312.0202)

[16] Tavallali P, Hou TY, Shi Z 2014 Extraction of intrawave signals using the sparse time–frequency representation method. SIAM Multiscale Model. Simul. 12, 1458–1493. (doi:10.1137/140957767)

[17] Pahlevan NM, Tavallali P, Rinderknecht DG, Petrasek D, Matthews RV, Hou TY, Gharib M 2014 Intrinsic frequency for a systems approach to haemodynamic waveform analysis with clinical applications. J. R. Soc. Interface 11, 20140617 (doi:10.1098/rsif.2014.0617) 25008087 10.1098/rsif.2014.0617 PMC4233710

[18] Pahlevan N, Rinderknecht D, Tavallali P, Petrasek D, Matthews R, Gharib M 2014 Intrinsic frequency method for noninvasive diagnosis of left ventricular systolic dysfunction. In Proc. 67th Annual Meeting of the APS Division of Fluid Dynamics, San Francisco, CA, 23–25 November 2014, vol. 59, abstract BAPS.2014.DFD.D7.6.

[19] Westerhof N, Lankhaar J-W, Westerhof BE 2009 The arterial windkessel. Med. Biol. Eng. Comput. 47, 131–141. (doi:10.1007/s11517-008-0359-2) 18543011 10.1007/s11517-008-0359-2

[20] Cooper LL, Rong J, Benjamin EJ, Larson MG, Levy D, Vita JA, Hamburg NM, Vasan RS, Mitchell GF 2015 Components of hemodynamic load and cardiovascular events. Circulation 131, 354–361. (doi:10.1161/CIRCULATIONAHA.114.011357) 25416177 10.1161/CIRCULATIONAHA.114.011357 PMC4308473

[21] Pahlevan NM, Gharib M 2011 Low pulse pressure with high pulsatile external left ventricular power: influence of aortic waves. J. Biomech. 44, 2083–2089. (doi:10.1016/j.jbiomech.2011.05.016) 21679951 10.1016/j.jbiomech.2011.05.016

[22] Pahlevan NM, Gharib M 2013 *In vitro* investigation of a potential wave pumping effect in human aorta. J. Biomech. 46, 2122–2129. (doi:10.1016/j.jbiomech.2013.07.006) 23915578 10.1016/j.jbiomech.2013.07.006

[23] Parragh S, Hametner B, Bachler M, Kellermair J, Eber B, Wassertheurer S, Weber T 2015 Determinants and covariates of central pressures and wave reflections in systolic heart failure. Int. J. Cardiol. 190, 308–314. (doi:10.1016/j.ijcard.2015.04.183) 25935618 10.1016/j.ijcard.2015.04.183

[24] Torjesen AA, Wang N, Larson MG, Hamburg NM, Vita JA, Levy D, Benjamin EJ, Vasan RS, Mitchell GF 2014 Forward and backward wave morphology and central pressure augmentation in men and women in the Framingham heart study. Hypertension 64, 259–265. (doi:10.1161/HYPERTENSIONAHA.114.03371) 24866142 10.1161/HYPERTENSIONAHA.114.03371 PMC4184952

[25] Tsao CW, Pencina KM, Massaro JM, Benjamin EJ, Levy D, Vasan RS, Hoffmann U, O’Donnell CJ, Mitchell GF 2014 Cross-sectional relations of arterial stiffness, pressure pulsatility, wave reflection, and arterial calcification. Arterioscler. Thromb. Vasc. Biol. 34, 2495–2500. (doi:10.1161/ATVBAHA.114.303916) 25169933 10.1161/ATVBAHA.114.303916 PMC4199901

[26] Pahlevan NM, Tran TT, Tavallali P, Rinderknecht DG, Csete M, Gharib MM 2015 New intrinsic frequency measures of cardiac function vs. cardiac MRI as a gold standard. In *ISMRM 23rd Annu. Meeting and Exhibition, Toronto, Canada, 30 May–5 June 2015*.

[27] Kolda TG, Lewis RM, Torczon V 2003 Optimization by direct search: new perspectives on some classical and modern methods. SIAM Rev. 45, 385–482. (doi:10.1137/S003614450242889)

[28] Boyd S, Vandenberghe L 2004 Convex optimization. Cambridge, UK: Cambridge University Press.

[29] Demmel JW 1997 *Applied numerical linear algebra*. Philadelphia, PA: Society for Industrial Mathematics.

[30] Bertsekas DP 1999 *Nonlinear programming*. Belmont, MA: Athena Scientific.

[31] Tavallali P, Hou TY, Rinderknecht DG, Pahlevan NM Data from: on the convergence and accuracy of the cardiovascular intrinsic frequency method. () 10.1098/rsos.150475 PMC4807454 27019733

